# Boy Bi! The Association Between HIV Knowledge, PrEP Stigma, Bisexual Contact, and Binegativity in a Sample of Undergraduate Students

**DOI:** 10.21203/rs.3.rs-10684472/v1

**Published:** 2026-09-28

**Authors:** Vincent Dang, Nathan Grant Smith

**Affiliations:** University of Houston; University of Houston

**Keywords:** HIV knowledge, binegativity, bisexual men, PrEP stigma, bisexual contact

## Abstract

**Background:**

Binegativity (negative attitudes and beliefs towards bisexual people) towards bisexual men includes inaccurate stereotypes of sexual promiscuity and serving as vectors of HIV to heterosexual women. College students’ HIV knowledge has been negatively associated with homonegative attitudes towards gay men; however, limited studies have explored the link between HIV knowledge and binegativity towards bisexual men. Like bisexual men, people who take HIV prevention medications (i.e., PrEP) are also stigmatized as being sexually irresponsible and promiscuous. However, limited studies have examined the link between PrEP stigma and binegativity towards bisexual men. To reduce binegativity, bisexual contact (positive and meaningful interactions with bisexual people) has been linked to reduced prejudice against bisexual people. The goal of the study was to examine the link between HIV knowledge, dimensions of PrEP stigma, bisexual contact, and binegativity towards bisexual men among undergraduate students (*N* = 633).

**Method:**

Undergraduate students were recruited through the University of Houston’s SONA system and answered self-report forms regarding demographic variables, bisexual contacts, HIV knowledge, dimensions of PrEP stigma, and attitudes towards bisexual men. Hierarchical multiple linear regression was used to test the relationship between HIV knowledge, dimensions of PrEP stigma, and bisexual contact with binegativity. Additionally, bisexual contact was tested as a potential moderator between HIV knowledge and binegativity, and between dimensions of PrEP stigma and binegativity.

**Results:**

HIV knowledge and bisexual contact were negatively associated with binegativity, while dimensions of PrEP stigma were positively associated with binegativity. Bisexual contact did not moderate the relationship between HIV knowledge and binegativity, and between dimensions of PrEP stigma and binegativity. These results suggest that LGBTQ inclusive sexual health education that includes accurate information about HIV, STIs, and PrEP may play a major role in reducing negative attitudes towards sexual minorities.

## Background

Binegativity refers to the marginalization and questioning of bisexuality as a valid identity, which often manifests in discrimination against bisexual people (1). Binegativity largely stems from inaccurate negative stereotypes towards bisexuals, such as bisexuals being untrustworthy, confused about their sexuality, less likely to be monogamous, and more likely to give a sexually transmitted infection (STI) to their partner (2). Negative views about bisexual men, specifically, include being less romantically attractive, less masculine, and less desirable as sexual partners compared to heterosexual men (3). Despite implicit stereotypical beliefs about bisexual men being prevalent, Zivony and Lobel (4) found that these beliefs are not explicitly acknowledged as stereotypes and people who were less knowledgeable about bisexual stereotypes had higher prejudice towards bisexual men. Research has shown that people have more positive attitudes towards bisexual women than bisexual men and that women generally hold more positive attitudes towards bisexual people than men (5, 6). Attitudes towards bisexual men are also significantly more negative than attitudes toward other sexual minorities (7). Common contributors to binegativity towards bisexual men include a lack of bisexual friends and acquaintances, belonging to a conservative religion, and being a man (8). Similarly, not being religious, being under the age of 30, knowing a bisexual person, and being lesbian/gay are associated with being less likely to socially distance oneself from bisexual people (9).

These inaccurate stereotypes and negative attitudes contribute to bisexual people facing unique stressors concerning their sexuality. First proposed by Brooks (10) and expanded by Meyer (11), the minority stress theory proposes that sexual minorities face higher rates of mental disorders due to various stress processes like prejudice events, identity concealment, and expectations of rejection. One example of bisexual specific minority stress is that bisexual people are subject to binegativity from both heterosexual and lesbian/gay communities (12–15). Additionally, Arriaga et al. (16) found that binegativity from lesbian and gay people, but not heterosexual people, was significantly associated with internalized binegativity. Bisexual specific minority stress is also affected by differences in their partner’s gender. Sarno et al. (17) found that bisexual men experienced more interpersonal hostility from heterosexuals, gays, and lesbians when their partner was a woman. Bisexual men who only had men sex partners were more likely to be stereotyped about not having a stable orientation by both heterosexuals, gays, and lesbians while men with only woman sex partners were more likely to be viewed as more sexually irresponsible by heterosexuals but not gay and lesbian people.

In an effort to reduce binegativity and concomitant negative mental health outcomes in bisexual men, researchers have looked to theories such as the intergroup contact theory to guide future research. Intergroup contact theory states that positive and meaningful interactions between people from different groups can reduce prejudice between them (18). Dyar et al. (19) suggests that intergroup contact between bisexuals and non-bisexuals could be associated with more positive attitudes towards bisexuals and less binegativity. Other researchers have implemented intergroup contact theory in their studies as a framework and found that having bisexual contacts reduced prejudice towards bisexuals (20, 21). Additionally, Lytle et al. (22) found knowing more bisexuals predicted more positive attitudes and decreased intergroup anxiety with bisexual men and women. Overall, intergroup contact may be beneficial in reducing prejudice and dispelling inaccurate stereotypes about bisexual men such as being hypersexual.

Because bisexual men are viewed as hypersexual and promiscuous, they are considered vectors of STI that are responsible for functioning like a “bridge” that passes on HIV and STIs to their unsuspecting partners (6, 23). Older published research has suggested that bisexual men are responsible for infecting their women partners with HIV (24, 25), but these studies have been refuted (26). A meta-analysis done by Friedman et al. (27) suggests that men who have sex with men and women likely present no greater risk of HIV transmission to women than exclusively heterosexual partners. Moreover, studies that suggest that bisexual men are a bridge of HIV transmission to women contain many methodological limitations (28). Furthermore, Sekuler et al. (29) found that sexually active bisexual individuals in heterosexual relationships reported the lowest percentage of sexual risk behaviors and were unlikely to play a role in HIV transmission into the general population.

As previously mentioned, one example of binegativity is that bisexual men are considered vectors of HIV and STI infection, suggesting that people still hold inaccurate HIV stereotypes and are lacking in HIV knowledge. Limited research to date has looked at the link between HIV knowledge and binegativity. However, previous studies have showcased that lack of knowledge about HIV plays a role in added stigma against sexual minority men (30–34). Additionally, college students who had less accurate information about HIV had higher levels of homonegativity while college students who had more accurate information about HIV had lower levels of homonegativity (35–38). Luchetta and Pardie (39) suggested that when people are lacking knowledge about HIV, stereotypical beliefs may be used to blame gay men to rationalize negative attitudes around HIV because HIV is (inaccurately) considered a disease that is exclusive to gay people. Because inaccurate stereotypes about bisexual men include being bridges of HIV to heterosexual women, using previous studies examining the relationship between HIV knowledge and homonegativity against gay men as a conceptual framework, the present study focused on the link between HIV knowledge and binegativity towards bisexual men.

Although most literature about HIV knowledge and stigma against sexual minority men was published before the year 2000, sexual minority men are still disproportionately affected by HIV, emphasizing the importance of this research. In 2022, male-to-male sexual contact made up 67% of new HIV infections in the U.S. (40). Additionally, general knowledge surrounding HIV is still lacking. According to the 2024 State of HIV Stigma Report, only 37% of Gen Z adults feel knowledge about HIV and 85% of Americans still feel there is a lingering stigma around HIV. Furthermore, the belief that people living with HIV can live long healthy lives has decreased since 2020 and only 67% of adults believe that they should get tested for HIV (41). Thus, given a lack of research on attitudes toward bisexual men and links with HIV knowledge, combined with continued high incidence of HIV in sexual minority men and low HIV knowledge in the general population, additional research into the intersection of binegativity and HIV knowledge is needed.

As noted, most of the research on HIV knowledge was published prior to 2000; however, in the years since, PrEP was introduced as a critical component of HIV prevention. When taken as prescribed, PrEP can safely reduce the incidence of HIV infection in high-risk individuals by more than 90% (42). Despite PrEP’s safety and efficacy, stigma regarding its use remains. PrEP stigma refers to the fear of being associated with the negative inaccurate stereotypes about PrEP users and PrEP itself (43–45). Like stereotypes about bisexual men, PrEP users are stereotyped as sexually irresponsible, which discourages PrEP interest and adherence due to the fear of partner rejection and being labeled as “promiscuous” (43, 46). Additionally, Calabrese (47) found that current PrEP interventions that focus on men who have sex with men (MSM) may be counterproductive as they perpetuate stigma and reinforce negative stereotypes. Another dimension of PrEP stigma involves expected disapproval by family, sex partners, and friends, which is associated with less PrEP interest and less comfort discussing PrEP with a provider (43). However, limited research has examined the links between aspects of PrEP stigma and binegativity. Because inaccurate stereotypes about bisexual men and PrEP users both include sexual promiscuity and associations with HIV, the present study examined the relationship between undergraduate students’ PrEP stigma and binegativity towards bisexual men.

Knowledge of PrEP has been positively associated with intention to use PrEP (48, 49) Furthermore, people who already know about PrEP have less stigma and have more positive attitudes towards PrEP (50). Because prior PrEP knowledge can be a confounding variable, including it as a covariate may help isolate the true effects of the dimensions of PrEP stigma on binegativity, minimizing the possibility that the observed effects are due to pre-existing differences in PrEP knowledge.

## Methods

### Research Questions and Hypotheses

The goal of this study was to examine the link between HIV knowledge, dimensions of PrEP stigma (PrEP-user stereotypes and expected disapproval by others), bisexual contact, and binegativity. Based on the literature, we developed our main research question: Do undergraduate students’ HIV knowledge, PrEP stigma, and bisexual contacts predict binegativity towards bisexual men? Using previous studies implementing intergroup contact theory to reduce binegativity as a framework, we asked participants about the quality and quantity of bisexuals they know to test if there is a moderating effect of bisexual contact on the relationship between HIV knowledge, dimensions of PrEP stigma, and binegativity towards bisexual men. Our independent variables were the scores on the HIV knowledge, PrEP stigma, and bisexual contact measures. Our dependent variable was the score on the binegativity measure.

The research questions were explored through the following hypotheses:

1. Participants’ HIV knowledge will be negatively correlated with binegativity towards bisexual men.
2. Participants’ PrEP stigma (PrEP-user stereotypes and PrEP disapproval by others) will be positively correlated with binegativity towards bisexual men.
3. Participants’ bisexual contact will be negatively correlated with binegativity towards bisexual men.
4. Higher levels of bisexual contact will moderate (i.e., strengthen) the relationship between HIV-knowledge and binegativity towards bisexual men, while higher levels of bisexual contact will moderate (i.e., weaken) the relationship between PrEP stigma (PrEP-user stereotypes and PrEP disapproval by others) and binegativity towards bisexual men.

For a conceptual model of the hypotheses, see Fig. 1.

## Procedure and Participants

The study received approval from the University of Houston’s Institutional Review Board (IRB# STUDY00005627). All participants provided informed consent via an online survey prior to their participation in the study. All data were collected and processed in accordance with the ethical standards of the American Psychological Association.

We recruited undergraduate college students through the University of Houston’s Sona system. Sona is an online system that allows researchers to recruit and manage potential undergraduate study participants. Eligible participants received course credit in exchange for their participation in the study. Using G-power (51), we calculated that we would need to recruit 103 participants to obtain power of .80 with a medium effect size (.15) and alpha = .05.

A total of 633 responses were received through SONA. A total of 172 responses were removed due to providing incomplete and insufficient information defined by not completing at least 90% of the survey (*n* = 73). Furthermore, other responses were removed for failing an attention check (*n* = 52) and suspected bots as indicated by duplicate responses and repeated IP addresses (*n* = 47). Therefore, a total of 461 responses were included in the final analysis. Female participants made up of 82.4% of our sample (*n* = 380), while male participants made up 17.6% (*n* = 81). Cisgender participants made up 95% of our sample (*n* = 437), while 5% of participants (*n* = 23) endorsed nonbinary gender identities or identities within the trans umbrella. The top two endorsed gender identities were cisgender woman (78.26%, *n* = 360) and cisgender man (16.74%, *n* = 77). Heterosexual participants made up over half of our sample (67.61%, *n* = 311), followed by bisexuals (18.26%, *n* = 84) and gays, lesbians, and others (14.13%, *n* = 65). Additionally, White participants made up just under half of our sample (42.5%, *n* = 196), followed by Asian (31.5%, *n* = 145), Black or African American (18.2%, *n* = 84), American Indian or Alaskan Native (3.3%, *n* = 15), and Native Hawaiian or Pacific Islander (1.7%, *n* = 8) participants. Non-Hispanic/Latino participants made up 59.9% of our sample (*n* = 276), while 36% endorsed Hispanic/Latino heritage (*n* = 166). Participants’ annual household income included $100,000 or more (28.4%, *n* = 131), followed by $25,000-$49,999 (16.3%, *n* = 75), less than $25,000 (12.4%, *n* = 57), $75,000-$99,999 (9.5%, *n* = 44), and $50,000-$74,999 (9.5%, *n* = 44).

## Measures

We asked demographic questions about the highest education level received, annual income, gender, and sexuality via self-report forms. We also asked participants about the quantity of bisexual people they know and the perceived quality of those relationships to answer our third research question. A standardized definition of bisexuality was given to the participants to ensure they could accurately fill out the survey, which was as follows: “bisexual people refer to those with the capacity for physical, romantic, and/or sexual attraction to more than one sex or gender.”

Binegativity towards bisexual men was measured using the Bisexualities: Indiana Attitudes Scale-Males (BIAS-m, (52)). The BIAS-m contains 26-items and uses a 5-point Likert-type scale ranging from 1 (*strongly disagree*) to 5 (*strongly agree*). Sample items include: “I think bisexual men are actually gay” and “When I think of bisexual men, I think of HIV.” Five positive items, (bisexual men can fall in love with both men and women, bisexual men are very sexy, bisexual men really know what they are doing in bed, most heterosexual women would like to date bisexual men, I think bisexuality for men is a legitimate sexual orientation) are reverse coded and reverse scored. The reported Cronbach’s alpha showed high internal consistency reliability with a score of .94. Cronbach’s alpha for this study similarly showed high internal consistency reliability with an alpha of .92. The BIAS-m is scored by summing up all the responses and subtracting the total number of items with higher scores indicating higher levels of binegativity towards bisexual men.

HIV knowledge was measured using a shortened version of the Brief HIV Knowledge Questionnaire (HIV-KQ-18; (53)). Correct responses have a value of 1 while incorrect answers or responding with “I don’t know” have a value of 0. A higher score indicates higher HIV knowledge. The HIV-KQ-18 has strong internal consistency with Cronbach’s alpha ranging between .75–.89. The HIV-KQ-18 also has strong test-retest reliability (*r* = .76–.94) and demonstrated construct validity as evidenced by non-significant relationships between the HIV-KQ-18 and other biasing constructs (e.g., social desirability) and a correlation of ≥ .90 with the previous HIV-KQ (53). The shortened version of the HIV-KQ-18 is used to reduce participant burden and includes 10 statements rather than the original 18 (54). Using the shortened version of the HIV-KQ-18 also allows the removal of outdated questions (e.g., “A person can get HIV by sitting in a hot tub or a swimming pool with a person who has HIV”). Sample items in the shortened version include “A person can get HIV from oral sex” and “Having sex with more than one partner can increase a person’s change of being infected with HIV” and “A woman can get HIV if she has anal sex with a man.” Participants can respond with “True,” “False,” or “I don’t know.” The Cronbach’s alpha for the shortened version showed moderate internal consistency reliability with a score of .76 (54). The Cronbach’s alpha for the present study had an internal consistency reliability at .79.

PrEP stigma was measured using the PrEP Anticipated Stigma Scale (PASS; (43)). The PASS contains eight items and demonstrates acceptable internal consistency with a Cronbach’s alpha of .76. The PASS also contains two subscales: the PrEP Disapproval by Others subscale (e.g., “My friends would approve of me taking PrEP”) and the PrEP-User Stereotypes subscale (e.g., “People would I assume I am gay if I took PrEP”). Both subscales demonstrated construct validity as evidenced by confirmatory factor analysis (43). The PrEP Disapproval by Others subscale and the PrEP-User Stereotypes subscale were both shown to have high internal consistency reliability with scores of .91 and .87, respectively. In this study, the PrEP Disapproval by Others subscale and the PrEP-User Stereotypes subscale demonstrated acceptable internal consistency reliability with scores of .81 and .79, respectively. The PrEP-User Stereotypes subscale contains five items and uses a four-point Likert-type scale ranging from 1 (*strongly disagree*) to 4 (*strongly agree*). The PrEP Disapproval by Others subscale contains three items and uses a four-point Likert-type scale that is reverse-scored ranging from 1 (*strongly agree*) to 4 (*strongly disagree*). Subscale scores are calculated by averaging across items. Higher subscale scores indicate stronger perceived disapproval by others for using PrEP and stronger perceived PrEP-User stereotypes. Prior to filling out the PASS, a brief definition and summary of PrEP was given to participants to ensure they can accurately fill out the questionnaire.

General descriptive knowledge about PrEP was collected using the PrEP Knowledge Questionnaire (55). The PrEP Knowledge Questionnaire is a non-validated questionnaire that contains six statements (e.g., “A person who uses PrEP no longer needs to be tested for HIV”) that participants can respond with “True,” “False,” or “I don’t know.” A higher percentage of answers answered correctly indicates higher general knowledge about PrEP. After this pre-assessment, a small description about PrEP and its uses was given to the participant to read, followed by the PrEP Anticipated Stigma Scale. This ordering allowed us to assess participants’ baseline knowledge (or lack of knowledge) about PrEP and control its potential influence on the relationship between dimensions of PrEP stigma and binegativity. Cronbach’s alpha internal reliability score for general descriptive knowledge about PrEP was high with a score of .87.

Finally, we asked participants about both the quality and quantity of bisexual men, women, and nonbinary contacts they know. For example, the quantity of self-reported bisexual contacts for bisexual men was measured by two items: “How many bisexual men do you know?” and “How frequently do you have contact with bisexual men?” The two items use a 5-point scale ranging from 1 (*none*) to 5 (*more than 10*) and 1 (*never*) to 5 (*very frequently*), respectively. These items were previously used to assess quantity of contacts with African immigrants (56) and have been adapted to assess quantity of contacts with bisexual men in our study. The scores of the two items were multiplied together to get one final score that represents the quantity of bisexual male contacts (56–58). The quality of bisexual contacts for bisexual men was measured by two items: “How well do you get along with the bisexual men you know and “How emotionally close do you feel to the bisexual men you know?” The two items use a 5-point scale ranging from 1 (*very poorly*) to 5 (*very well*) and 1 (*very distant*) to 5 (*very close*), respectively. These items were previously used to assess quality of contact between grandchildren and their grandparents (59) and have been adapted to assess the quality of contact with bisexual men in our study. The scores of the two items were added together to get one final score that represents the quality of bisexual male contacts. Both scales were found to have acceptable internal consistency reliability with a score of .72 for quantity of contact and a score of .67 for quality of contact (56). In the present study, the internal consistency reliability for quantity of bisexual contacts was poor with a score of .42, while the internal consistency reliability for quality of bisexual contacts was acceptable with a score of .69.

Consistent with use in other studies, the two quality and quantity bisexual contact scores were then multiplied together to create one final index score of bisexual contact that considers both aspects of contact simultaneously, with higher scores resembling more frequent and positive contact (56, 57).

## Statistical Analyses

We used multiple linear regression via SPSS to test the direct relationship between our four predictor variables (HIV knowledge, PrEP-user stereotypes, PrEP disapproval by others, bisexual contact) and our outcome variable (binegativity). We also tested the moderating effect of bisexual contact (the single index score that simultaneously represents the quality and quantity of bisexual contacts) on the relationship between HIV knowledge, PrEP-user stereotypes, PrEP disapproval by others, and binegativity. As such, there were four predictor variables (HIV knowledge, PrEP-user stereotypes, PrEP disapproval by others, bisexual contact), one moderator (bisexual contact), and three interaction terms (HIV knowledge X bisexual contact, PrEP-user stereotypes X bisexual contact, and PrEP disapproval by others X bisexual contact). PrEP knowledge was included as a covariate. Additionally, we tested gender and sexuality differences to control for potential significant differences in the main analyses.

## Results

Before the test of the main analyses, descriptive statistics and bivariate analyses were calculated. If participants completed 80% of a scale, then mean scores were calculated for them. For any scales that required a sum score, the mean was multiplied by the number of items. A one-way ANOVA was conducted to compare the effect of gender (men, women, transgender/nonbinary) on binegativity. The results of the ANOVA revealed that there was a statistically significant difference in binegativity between groups *F*(2,457) = 11.67, *p* < .001). Tukey’s HSD test for multiple comparisons found that the mean value of binegativity was significantly (*p* < .001) different between transgender/nonbinary students (*M* = 13.11, *SD* = 12.98) and both men (*M* = 32.03, *SD* = 17.12) and women (*M* = 27.67, *SD* = 16.55). There was no statistical difference in mean scores of binegativity between men and women. Due to the overall statistical significance between groups, the trichotomous gender variable was collapsed into a new dichotomous variable (0 = transgender/nonbinary students, 1 = cisgender students) and added into the model as a covariate. A one-way ANOVA was also conducted to compare the effect of sexuality (heterosexuals, bisexuals, gays/lesbians/others) on binegativity. The results of the ANOVA revealed that there was a statistically significant difference in binegativity between groups *F*(2,457) = 40.31, *p* < .001. Tukey’s HSD test for multiple comparisons found that the mean value of binegativity was significantly different (*p* < .001) between heterosexual (*M* = 32.22, *SD* = 16.92) and both bisexual (*M* = 17.74, *SD* = 12.28) and gay/lesbian/other students (*M* = 19.03, *SD* = 12.38). There was no statistical difference in mean scores of binegativity between bisexual and gay/lesbian/other students. Like gender, the sexuality variable was collapsed into a new dichotomous variable (0 = sexual minority students, 1 = heterosexual students) and added into the model as a covariate. See Table 1 for bivariate correlations among study variables.

Tests of multicollinearity were performed with no violations. Hierarchical multiple regression moderation analysis was conducted to assess the moderating role of bisexual male contacts on the relationship between HIV knowledge, PrEP-user stereotypes, PrEP disapproval by others, and binegativity. See Table 2. The first block of the regression included PrEP knowledge, sexuality, and gender as covariates. This block significantly predicted binegativity (*R*^2^ = .15, Adj. *R*^2^ = .14, *F*(3, 309) = 17.40, *p* < .001). Sexuality (*p* < .001) emerged as a significant covariate. In the second block, the following mean-centered predictors were added to the model: HIV knowledge, PrEP-user stereotypes, PrEP-disapproval, and bisexual contact. The model continued to significantly predict binegativity (*R*^2^ = .28, Adj. *R*^2^ = .27, *F*(7, 305) = 17.10, *p* < .001) and had a significant *R*^2^ change of .14, *p* < .001. Sexuality remained a significant covariate (*p* < .001). HIV knowledge (*p* < .01), PrEP-user stereotypes (*p* < .001), PrEP-disapproval by others (*p* < .01), and bisexual contact (*p* < .001) all emerged as significant predictors. The final block included the following moderation interaction terms: HIV knowledge X bisexual contact, PrEP-user stereotypes X bisexual contact, and PrEP-disapproval by others X bisexual contact. The overall model continued to significantly predict binegativity (*R*^2^ = .29, Adj. *R*^2^ = .27, *F*(10, 302) = 12.31, *p* < .001), although the *R*^2^ change of .01 was not significant (*p* = .35). Sexuality remained a significant covariate (*p* < .001). Additionally, HIV knowledge (*p* = .01), PrEP-user stereotypes (*p* < .001), PrEP-disapproval by others (*p* < .01), and bisexual contact (*p* < .001) continued to be significant predictors. However, none of the interaction terms were significant. These results suggest that bisexual contact does not alter the relationship between HIV knowledge, dimensions of PrEP stigma, and binegativity. However, HIV knowledge, PrEP stigma, and bisexual contact were all significantly related to binegativity. Specifically, being more knowledgeable about HIV and having more bisexual contact was associate with less binegativity towards bisexual men. Additionally, higher levels of PrEP stigma were associated with more binegativity towards bisexual men. Given that bisexual participants might interpret questions about binegativity towards bisexual men differently than heterosexual and gay/lesbian participants (e.g., internalized binegativity), the statistical analyses were re-run without the inclusion of bisexual participants, and the pattern of results was not different (data not shown).

## Discussion

The goal of this study was to understand the relationship between college students’ HIV knowledge, dimensions of PrEP stigma, and bisexual contacts with binegativity towards bisexual men. The study also attempted to examine if the bisexual contact strengthened the relationship between HIV knowledge and binegativity towards bisexual men and weaken the relationship between dimensions of PrEP stigma and binegativity towards bisexual men in a sample of college students. To the authors’ knowledge, this research study is the first of its kind to examine the links between HIV knowledge and binegativity. The findings from this study support our hypotheses that HIV knowledge is significantly negatively correlated with binegativity towards bisexual men. These findings support previous literature indicating similar negative associations between HIV knowledge and homonegativity towards gay men (35–38). Because bisexual men are inaccurately stereotyped for being vectors of HIV, this study also supports previous research that having higher HIV knowledge is associated with less HIV-related stigma and discrimination towards MSM (31). Additionally, this study found that having more frequent and more positive relationships with bisexual men was correlated with less binegativity towards bisexual men. This finding supports similar past bisexual contact research (19–22).

Because most research around HIV knowledge was published prior to 2000, this study also provides updated information about newer HIV prevention methods like PrEP, as well as dimensions of PrEP-related stigma and their relationship with binegativity. The findings from this study support our hypothesis that higher levels of PrEP stigma are associated with higher levels of binegativity towards bisexual men. Although there is limited literature examining the relationship between PrEP stigma and binegativity specifically, this finding supports similar domains of research where higher levels of PrEP stigma was associated with higher levels of internalized homonegativity (60), external homonegativity (61), and HIV stigma towards MSM (46).

Previous studies have suggested intergroup contact can reduce prejudice by moderating or “weakening” the effects of intolerant social norms and intergroup anxiety (62, 63). Therefore, we predicted that bisexual contact would also moderate the relationship between HIV knowledge and binegativity (i.e., the impact of HIV knowledge on binegativity towards bisexual men might be stronger for those with more bisexual contact) and between PrEP stigma and binegativity (i.e., the impact of PrEP stigma on binegativity towards bisexual men might be weaker for those with more bisexual contact). However, our moderation results were not significant. This may be explained by bisexual contact being insignificantly correlated with HIV knowledge (*r* = .11, *p* = .05) and PrEP-user stereotypes (*r* = − .06, *p* = .30).

Conceptually, having more bisexual contact may fail to moderate (i.e., strengthen) the relationship between HIV knowledge and binegativity, even when a person has high HIV knowledge, because binegativity is typically rooted in deeply ingrained social stigmas (e.g., bisexuals being inaccurately stereotyped for being disproportionately sexually promiscuous and confused about their sexuality) rather than a lack of factual information (2). Simply knowing and having good relationships with bisexual men may not challenge this for someone, as they may view those men as exceptions to the rule. As previously stated in the introduction, bisexual men face dual discrimination from both heterosexual and lesbian/gay communities which may create a widespread cultural environment that sustains prejudice regardless of an individual’s personal interactions (12–15). Additionally, the rigid belief of monosexism (the belief that people can only be attracted to one sex or gender) is still pervasive in society (64). Therefore, more frequent and positive bisexual contact may not automatically dismantle underlying structural biases against bisexuality.

While binegativity often stems from binary views of sexuality and social stigmas, PrEP stigma is more heavily tied to moral judgments about sexual promiscuity, risk-taking, and infidelity (65, 66). Because these stigmas stem from different belief systems, increasing bisexual contact may not change judgments about PrEP and PrEP users. This may explain why bisexual contact failed to significantly moderate (i.e., weaken) the relationship between dimensions of PrEP stigma and binegativity in our study and was not significantly associated with PrEP-user stereotypes. Despite this, bisexual contact was still significantly correlated with PrEP knowledge (*r* = .20, *p* < .001), PrEP disapproval by others (*r* = − .12, *p* < .05) and binegativity (*r* = − .31, *p* < .001) in the present study at the bivariate level.

Clinical implications based on the results from this study suggest that efforts to reduce binegativity towards bisexual men include providing more accurate and updated sexual education about HIV and PrEP and combatting inaccurate stereotypes towards sexual minorities. This is evidenced by the low scores of college students’ HIV knowledge (*M* = 5.6, *SD* = 2.8, maximum possible score = 10) and PrEP knowledge (*M* = 1.8, *SD* = 2.0, maximum possible score = 6) in this study. Additionally, Manalastas et al. (67) found that interventions combining positive bisexual contact and multicultural education and may be the most effective approach to reducing binegativity. This can be facilitated through promoting positive interactions with bisexual people via in-person encounters that develop into friendships (68), promoting positive interactions with bisexual people online (69), and implementing multicultural education that integrates accurate information about bisexual lives, history, and experiences with current sexual health education. This is especially important as current sexual health education rarely includes LGBTQ people’s experiences (70, 71). Therefore, LGBTQ inclusive comprehensive sexual health education that integrates accurate information about HIV/STIs can help reduce negative attitudes towards bisexual men and other sexual/gender minorities. A final approach that Manalastas et al. (67) suggest is using cognitive approaches to challenge rigid and outdated beliefs about human sexuality (e.g., there are only two sexualities: gay and straight). These cognitive approaches may also be used to challenge inaccurate stereotypes about bisexual men (such as being vectors of HIV/STI) and can be integrated along with LGBTQ inclusive sexual health education.

## Limitations & Strengths

One general limitation of the current study is the recruitment of White, educated, industrialized, rich, and democratic participants, who are common among studies involving college students (72). As previously noted in the methods section, our sample was primarily made up of students that were cisgender women, heterosexual, White, non-Hispanic, and had a household income of $100,000 or more. Although college students were our population of interest, college student samples in general may impact the generalizability of the study to the broader population. Additionally, the self-selection of enrolling in studies through the SONA portal may have caused implicit bias in our study. For example, students may have been more likely to volunteer to take part in this study due to holding certain viewpoints about the research topics, which may have affected results. Future studies should address this limitation by using random sampling and recruiting samples outside of college students.

Another limitation of the proposed study is the lack of current validated HIV and PrEP measures for the general population. Most HIV and PrEP measures in the literature are tailored towards MSM, gender diverse populations, and medical professionals. However, the measures for the general population tend to ask outdated questions (e.g., “A person can get HIV from a toilet seat,”(73)) and do not reflect the current scope of medical advancement of HIV and PrEP research (e.g., post-exposure prophylaxis medication to prevent HIV infection after possible exposure or long-acting injectable PrEP). We used the 10-item shortened version of the HIV-KQ-18 that removed outdated questions to address this concern. Researchers should develop up-to-date validated measures for HIV and PrEP knowledge that can be tailored towards the general population and at-risk communities.

Another limitation of this study is the low Cronbach’s alpha for the measure of quantity of bisexual contacts (*a* = .42). This low score may be due to the scale consisting of only two questions. However, this scale is consistent with what has been used in previous intergroup contact research (56). Ultimately, it was decided to keep the quantity of contact scale, as an optimal combination of both quantity and quality of contact are needed to reduce prejudice (18). In general, low Cronbach’s alpha can be addressed by increasing the number of related items and/or re-running reliability analyses after removing items with low item-total correlations.

In addition to limitations, the present study contains strengths as well. As noted earlier, this study had a large sample size (*N* = 461), indicating strong power. Additionally, we studied binegativity towards bisexual men, which has been an understudied population in social science research (74). This study also adds to the intergroup contact literature by simultaneously measuring both the quantity and quality of bisexual contacts, and its potential effects as a moderator. Finally, this research study addresses important gaps in literature by examining the links between HIV knowledge and PrEP stigma with binegativity.

## Conclusion

Overall, this study added to the sexual health and sexual minority literature by examining relationships between HIV knowledge, PrEP stigma, bisexual contact, and binegativity. Although the quality and quantity of bisexual contacts did not moderate the relationship between HIV knowledge, PrEP stigma, and binegativity towards bisexual men as hypothesized, we found that HIV knowledge and more frequent positive bisexual contact was significantly associated with less binegativity towards bisexual men, while higher levels of PrEP stigma was significantly associated with higher levels of binegativity towards bisexual men. These findings suggest that LGBTQ inclusive sexual health education that integrates accurate and updated information about HIV, STIs, and PrEP may play a major role in reducing negative attitudes towards sexual minorities.

## Figures and Tables

**Figure 1:**
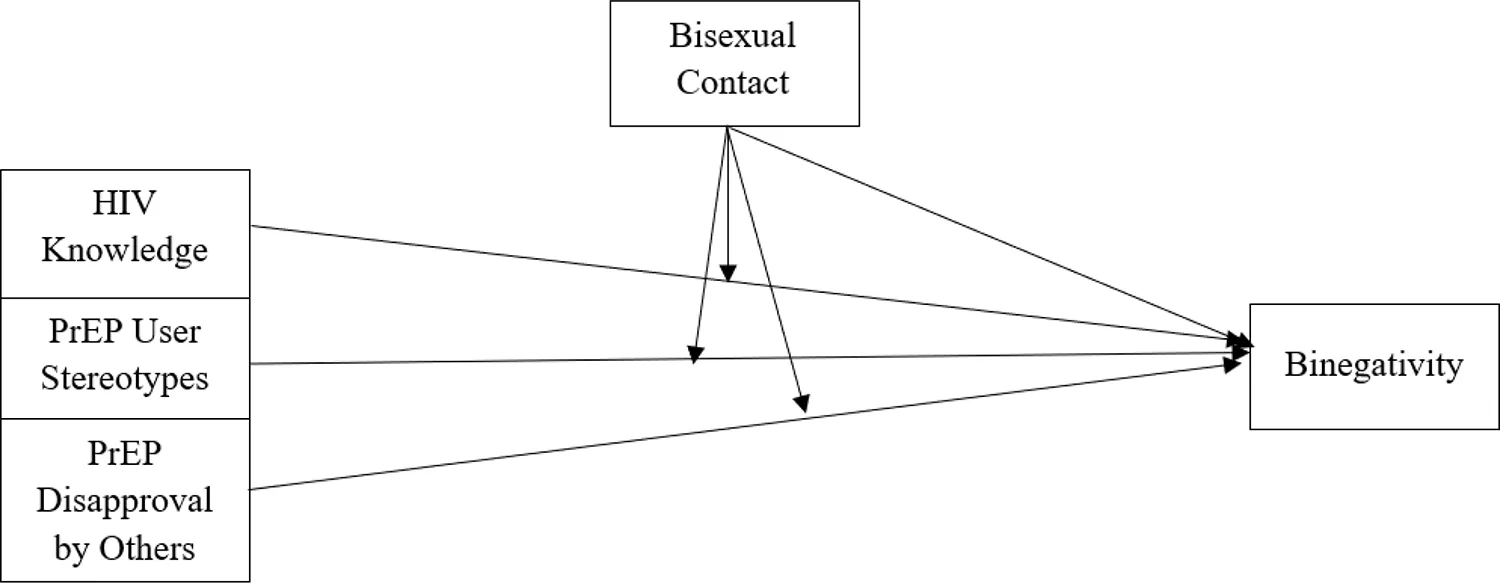
Conceptual Moderation Model

**Table 1:** Correlation Matrix

Variable	1	2	3	4	5	6	7	8
1. Gender^a^	-	-	-	-	-	-	-	-
2. Sexuality^b^	.31***	-	-	-	-	-	-	-
3. PrEP Knowledge	−.05	−.13**	-	-	-	-	-	-
4. HIV Knowledge	−.05	− .07	.48***	-	-	-	-	-
5. PrEP User Stereotypes	−.01	.05	.06	.1*	-	-	-	-
6. PrEP Disapproval by Others	.09	.14**	−.1*	−.09	.24***	-	-	-
7. Bisexual Contact	−.13*	−.27***	.20***	.11	−.06	−.12*	-	-
8. Binegativity	.20***	.39***	−.15**	−.15**	.27***	.28***	−.31***	-

Note.

* p < .05,

** p < .01,

*** p < .001.

a Transgender/nonbinary students = 0, cisgender students = 1.

b Sexual minority students = 0, heterosexual students = 1.

**Table 2:** Hierarchical Linear Regression Results

Variable	*B*	*SE*	β	*R* ^2^	Δ*R*^2^
Block 1				.15	.15***
Constant	13.92***	3.34			
PrEP Knowledge	−.58	.43	−.07		
Gender^a^	6.92	3.52	.11		
Sexuality^b^	10.57***	1.92	.31***		
Block 2				.28	.14***
Constant	15.37***	3.12			
PrEP Knowledge	.30	.50	.04		
Gender^a^	5.55	3.26	.09		
Sexuality^b^	7.99***	1.82	.24***		
HIV Knowledge	−.94*	.34	−.16*		
PrEP User Stereotypes	5.32***	1.38	.20***		
PrEP Disapproval by Others	3.68**	1.25	.15**		
Bisexual Contact	1.0***	.03	−.20***		
Block 3				.29	.01
Constant	14.78	3.17			
PrEP Knowledge	.30	.46	.04		
Gender^a^	5.96	3.31	.09		
Sexuality^b^	7.93***	1.83	.23***		
HIV Knowledge	−.88*	.34	−.15*		
PrEP User Stereotypes	5.16***	1.38	.19***		
PrEP Disapproval by Others	3.68**	1.26	.15**		
Bisexual Contact	−.10***	.03	−.21***		
HIV Knowledge X Bisexual Contact	.01	.01	.07		
PrEP Stereotypes X Bisexual Contact	−.03	.05	−.03		
PrEP Disapproval by Others X Bisexual Contact	−.02	.04	−.03		

*Note*:

* *p* < .05,

** *p* < .01,

*** *p* < .001.

a Transgender/nonbinary students = 0, cisgender students = 1.

b Sexual minority students = 0, heterosexual students = 1.

## Data Availability

The datasets used and/or analyzed during the current study are available from the corresponding author on reasonable request

## Abbreviations

STI: Sexually transmitted infection
MSM: Men who have sex with men
BIAS: m–Bisexualities: Indiana Atittudes Scale–Males
HIV: KQ–18–HIV Knowledge Questionnaire 18
PASS: PrEP Anticipated Stigma Scale
